# Cuproptosis in stroke: focusing on pathogenesis and treatment

**DOI:** 10.3389/fnmol.2024.1349123

**Published:** 2024-03-28

**Authors:** Liwei Xing, Zhifeng Wang, Zhihui Hao, Pan Pan, Aiming Yang, Jian Wang

**Affiliations:** ^1^The First Clinical Medical School, Yunnan University of Traditional Chinese Medicine, Kunming, Yunnan, China; ^2^College of Acupuncture and Massage, Yunnan University of Traditional Chinese Medicine, Kunming, Yunnan, China; ^3^Yunnan Provincial Hospital of Traditional Chinese Medicine, Kunming, Yunnan, China

**Keywords:** stroke, cuproptosis, pathogenesis, treatment, mechanism

## Abstract

Annually, more than 15 million people worldwide suffer from stroke, a condition linked to high mortality and disability rates. This disease significantly affects daily life, impairing everyday functioning, executive function, and cognition. Moreover, stroke severely restricts patients’ ability to perform daily activities, diminishing their overall quality of life. Recent scientific studies have identified cuproptosis, a newly discovered form of cell death, as a key factor in stroke development. However, the role of cuproptosis in stroke remains unclear to researchers. Therefore, it is crucial to investigate the mechanisms of cuproptosis in stroke’s pathogenesis. This review examines the physiological role of copper, the characteristics and mechanisms of cuproptosis, the differences and similarities between cuproptosis and other cell death types, and the pathophysiology of cuproptosis in stroke, focusing on mitochondrial dysfunction and immune infiltration. Further research is necessary to understand the relationship between previous strokes and cuproptosis and to clarify the mechanisms behind these associations.

## 1 Introduction

Annually, stroke affects approximately 15 million people globally, leading to high mortality and disability rates. Recent statistics rank stroke as the second leading cause of death worldwide (Campbell et al., 2020; Krishnamurthi et al., 2020). Stroke patients often suffer structural brain damage due to a combination of ischemia and bleeding (Zhang et al., 2021). Even a mild stroke can significantly impact various aspects of an individual’s daily functioning, including executive function and cognition (Fride et al., 2015). Various pathways have been identified in stroke-induced damage, such as mitochondrial dysfunction, ferroptosis, and apoptosis. Recently, a novel process called cuproptosis has been proposed as a significant contributor to stroke development. However, the specific factors involved in cuproptosis-related stroke are not yet fully understood. This review delves into the physiological role of copper, the characteristics and mechanisms of cuproptosis, the distinctions and parallels between cuproptosis and other cell death forms, and the pathophysiology of cuproptosis in stroke.

## 2 Fundamental aspects of cuproptosis

### 2.1 Physiological function of copper

Copper exists in two ionic states within living organisms: the reduced copper ion (Cu^+^) and the oxidized copper ion (Cu^2+^), due to its REDOX activity (Haitao et al., 2023). These ions can bind with various proteins or enzymes, serving as cofactors or structural components. They participate in regulating physiological processes including energy metabolism, mitochondrial respiration, and antioxidation (Rui et al., 2023). Additionally, copper plays a role in key biological processes, including angiogenesis, hypoxia response, and neuromodulation (Tianwen et al., 2023). It is involved in the generation of reactive oxygen species (ROS) and the oxidative modification of low-density lipoprotein cholesterol (Huiqin et al., 2023). Furthermore, copper impacts the nervous system by contributing to myelination, synaptic activity regulation, signaling cascades, and neuronal death modulation (Rui et al., 2023).

### 2.2 Role of trace metal elements in the central nervous system

Alterations in the levels of essential metal elements, such as iron, zinc, and copper, signal the onset of various neurological diseases (Ayton et al., 2013). Iron and its associated proteins, vital for brain development, are abundantly present in the brain. They regulate the synthesis and release of nerve myelin and dopamine neurotransmitters. Iron deficiency in the brain impairs ganglia function, inhibits nerve impulse conduction, and affects brain development and function, leading to decreased attention, memory, and motor coordination (Acevedo et al., 2019). Zinc, as the active center of many enzymes, participates in hormone secretion and bone calcification regulation. It is involved in the synthesis of enzymes and neurotransmitters in brain tissue and is associated with various neurodegenerative diseases. Both excessive and insufficient zinc levels can increase disease risk (Li et al., 2022). Copper, crucial for heme synthesis, immune function, and nerve conduction, is an essential transition metal in the human body. It is involved in oxygen metabolism, collagen synthesis, iron homeostasis, and antioxidant defense. In the nervous system, copper contributes to myelin formation, synaptic activity regulation, neuron death, and signal cascade reaction. Maintaining copper metabolism homeostasis is vital (Rui et al., 2023). Additionally, cerium plays a significant role in the nervous system. It can stimulate human growth, balance and regulate metabolism, and influence growth, reproductive function, brain development, and aging. The cerium content in the adult brain decreases with age (Roberts et al., 2016).

### 2.3 Characteristics of cuproptosis

Cuproptosis is a newly discovered form of cell death that occurs when there is an imbalance in the regulation of copper ions within cells. These ions can directly interact with fatty acylated components in the tricarboxylic acid cycle (TCA), leading to the aggregation and imbalance of certain proteins, which ultimately disrupts the TCA cycle. This disruption results in toxic stress on proteins and ultimately causes cell death (Tianwen et al., 2023). The process begins with a reduction in the lipoylation of DLAT and DLST, as well as the formation of oligomers of lipoylated proteins involved in the TCA cycle, due to the direct binding of copper. Additionally, there is a destabilization and overall decrease in iron–sulfur (Fe-S) cluster proteins. These effects combine to induce proteotoxic stress and impair mitochondrial function (Huo et al., 2023). Another critical process is the necessity of fatty acylation for binding copper ions. The binding of copper ions to fatty acylated DLAT and the subsequent induction of oligomerization are noted. Moreover, the harmful synthesis of fatty acylated proteins upon exposure to a copper ionophore is partly mediated by aberrant oligomerization. Additionally, the depletion of the endogenous intracellular copper chaperone glutathione leads to copper-dependent cell death (Huiqin et al., 2023). This phenomenon is associated with a reduction in fatty acylation due to the weakening of FDX1 and low-intensity aerobic exercise (LIAS), as well as an increase in DLAT oligomerization (Jia et al., 2023).

### 2.4 Mechanism of cuproptosis

**Figure 1:**
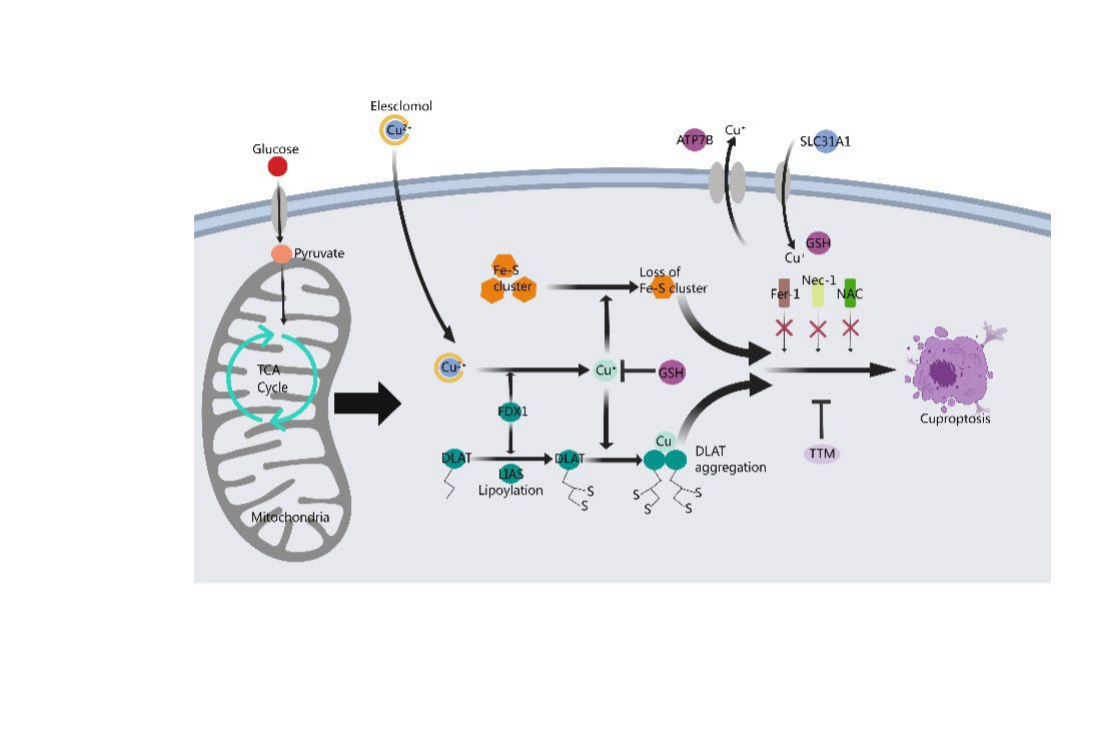
Mechanism of cuproptosis. A novel cell death process called cuproptosis provides strong evidence for the concept that copper-induced cell death occurs through a well-established mechanism involving protein lipoacylation. Cuproptosis begins with copper directly interacting with the fatty acylated parts of the tricarboxylic acid (TCA) cycle. This interaction causes the accumulation of fatty acylated proteins and eventually disrupts iron–sulfur cluster proteins, leading to proteotoxic stress and resulting in cell death. Copper chelator TTM inhibited cuproptosis, while ferroptosis inhibitor (Fer-1), necrotizing apoptosis inhibitor (Nec-1) and oxidative stress inhibitor (NAC) had no effect on cuproptosis. FDX1 acts as a key regulator of protein lipoacylation, which is essential for controlling cuproptosis. Both FDX1 and protein lipoacylation are critical in managing this cell death process.

Cuproptosis disrupts mitochondrial respiration and protein lipidation, leading to membrane permeability, cellular damage, and the initiation of apoptotic pathways under various conditions (Chen et al., 2022). Firstly, copper induces harm through oxidative stress and facilitates cell death, with oxidative stress being a primary inducer of apoptosis by impairing fundamental biomolecules such as proteins, lipids, and DNA. Additionally, the presence of copper may amplify the effects of oxidative stress (Acilan et al., 2016). Secondly, protein acylation triggered by copper ultimately leads to cell death. High concentrations of copper ions can interfere with the normal configuration and operational mechanisms of proteins due to their affinity for protein binding sites. Copper targeting and binding to fatty acylated constituents of the TCA cycle result in the aggregation of these Cu-bound fatty acylated proteins within the mitochondria, leading to a reduction of iron–sulfur (Fe-S) clusters and triggering proteotoxic stress, ultimately causing cell death. Thirdly, the breakdown of copper homeostasis mediates cell death (Cobine and Brady, 2022). It is crucial to maintain an adequate concentration of copper ions within the cell for normal physiological metabolism and to implement precautionary measures to prevent excessive uptake of copper ions, whose toxicity can lead to cell death. Lastly, cell death triggered by copper ionophores occurs following an accumulation phase of intracellular copper ions, facilitating the anomalous oligomerization of lipoacylated proteins in the TCA cycle and leading to a decrease in iron–sulfur cluster levels. Copper ionophores can transport copper ions into cells, thereby elevating the intracellular Cu^2+^ concentration, promoting the generation of reactive oxygen species (ROS), impeding the activity of the proteasome, and ultimately triggering cell death (Figure 1).

### 2.5 Similarities and differences between cuproptosis and other cell death modes

Ferroptosis is a form of cell death dependent on iron, where an excessive iron accumulation leads to the buildup of lipid peroxides in cellular membranes, resulting in programmed cell death (Jiang et al., 2021). When comparing ferroptosis and cuproptosis, it is clear that mitochondria play a critical role in both types of cell death. Dihydrolipamide S-acetyltransferase (DLAT) (Liu et al., 2024) is central to this process. Copper directly binds to DLAT, promoting the formation of disulfide bonds in fatty acylated DLAT, which triggers cell death. This process can be mitigated by mitochondrial glutathione (GSH), which inhibits lipid acylation of the enzyme and promotes DLAT oligomerization. In ferroptosis, using mitoquinone (MitoQ), a mitochondria-targeting compound, has been shown to increase GSH levels, maintaining mitochondrial integrity and protecting cells from lipid peroxide accumulation, thus preventing cell death. Contrarily, ferroptosis involves mitochondrial morphological changes, such as shrinkage, increased membrane density, and mitochondrial fragmentation (Li J. et al., 2020; Li X. et al., 2020), not observed in cuproptosis. Further research is needed to identify the specific morphological features of cuproptosis and its potential link to iron homeostasis disruption and ferroptosis induction.

Apoptosis is primarily triggered by physiological or minor pathogenic stimuli and is an active cellular demise mechanism (Elmore, 2007), occurring without any inflammatory response. It is characterized by distinct cellular and tissue alterations, different from necrosis.

Necrosis is caused by pathogenic stimuli or severe injury, leading to the release of cellular contents outside the cell and subsequent inflammation (Nikoletopoulou et al., 2013). It features cell swelling, membrane disruption, content leakage, nuclear changes, partial DNA destruction, and significant local inflammatory responses. In the context of pyroptosis (Fang et al., 2020), activated cysteine aspartate proteases cleave GSDMD/GSDME into fragments that form pores in the cellular membrane, releasing inflammatory cytokines and other cell components. Apoptosis, pyroptosis, and necrosis are distinct cell death mechanisms that together create a cohesive system, where pathways can compensate for each other. These pathways might be activated sequentially in a single cell, depending on time and environmental factors. It is acknowledged that cell death is not caused by a single mechanism. Instead, there is a reciprocal interaction between pathways, where modulation of one can significantly affect the others (Ketelut-Carneiro and Fitzgerald, 2022).

Autophagy is a cellular breakdown mechanism conserved across evolution and tightly regulated in eukaryotic cells. It activates in response to metabolic stimuli, either internal or external, such as nutrient deprivation or low oxygen levels. This mechanism involves the sequestration and delivery of cellular components including aggregates, excessive or damaged organelles, and pathogens, to specialized compartments called lysosomes. Within the lysosomes, these components undergo hydrolysis, breaking down into smaller molecules, such as nucleotides, which are then recycled back into the cytoplasm for utilization by the cell (Li J. et al., 2020; Li X. et al., 2020).

## 3 Pathogenesis of cuproptosis in stroke

### 3.1 Cuproptosis is associated with brain damage

Cuproptosis, a relatively new concept in cellular death, has recently been explored in several scholarly studies, particularly for its link to cerebral impairment. Patel’s research provides experimental evidence of a correlation between brain copper imbalance, neurotoxicity, and the development of Alzheimer’s disease (Patel and Aschner, 2021). However, other studies suggest that copper presence in the brain might protect against cognitive decline in individuals with Alzheimer’s disease (Agarwal et al., 2022). Research has also indicated that dietary copper restriction, the use of copper chelators, and genetic manipulation of copper transporters could slow Huntington’s disease progression in animal models (Hands et al., 2010). In amyotrophic lateral sclerosis (ALS) (Gil-Bea et al., 2017), difficulties arise in the interaction between the copper chaperone of superoxide dismutase (CCS) and mutant copper-zinc superoxide dismutase (SOD1). This impairment in interaction leads to reduced copper delivery to mitochondria, accumulation of unstable SOD1, and increased susceptibility to pro-oxidants. These molecular events result in toxic gain-of-function effects, particularly in motor neurons. The study also indicates that SOD1 mitigates neuronal damage following cerebral ischemia by decreasing the release of mitochondrial cytochrome C and inhibiting caspase activation. Gliomas, originating from abnormal glial cells or their precursors, represent a common and deadly type of primary intracranial malignancy (Xu et al., 2020). Cell death within tumors often occurs due to metabolic stressors like hypoxia or glucose deprivation (Qin et al., 2019). Consequently, cuproptosis could be a uniquenovel therapeutic approach for cancer treatment (Wang T. et al., 2022; Wang W. et al., 2022). Studies suggest that prolonged occupational exposure to copper increases the risk of developing Parkinson’s disease, with excess copper inducing neuronal cell death and α-synuclein aggregation (Montes et al., 2014). Parkinson’s disease (PD) progression may also be linked to mitochondrial malfunction and oxidative stress (Vallée et al., 2020).

### 3.2 Stroke and cuproptosis related mitochondrial dysfunction

Fang Huiqin hypothesized that mitochondria are vital for cellular homeostasis, with disruptions often resulting from reduced mitochondrial DNA copy number and oxygen consumption (Huiqin et al., 2023). Therefore, treating AS involves regulating copper ion homeostasis and improving mitochondrial dysfunction to affect vascular endothelial function and macrophage polarization, reducing stroke risk. Previous research supports the link between mitochondrial dysfunction and decreased adenosine triphosphate (ATP) synthesis in ischemic strokes, leading to cerebral cell death due to ATP deficiency. However, given cuproptosis depends on mitochondrial respiration and the tricarboxylic acid cycle, it’s hypothesized to be a key mechanism in ischemia and hypoxic nerve cell and oligodendrocyte death. Still, conclusive evidence for this is currently lacking (Wang T. et al., 2022; Wang W. et al., 2022). Huo et al. (2023) and Jia et al. (2023) described cuproptosis as initiated by the nonspecific interaction between copper and fatty acylation proteins in mitochondria. This interaction reduces the expression of proteins in the mitochondrial oxidative respiratory chain, decreasing the activity of mitochondrial complexes I and III, and lowering ATP content. This leads to cellular energy deficits and cardiomyocyte death. Considering the high oxygen and ATP demands of brain tissue, it is worth exploring whether cuproptosis, known to cause cardiac cell death, could also play a role in stroke development. However, this theory needs further validation.

### 3.3 Stroke and cuproptosis-related immune infiltration

**Figure 2:**
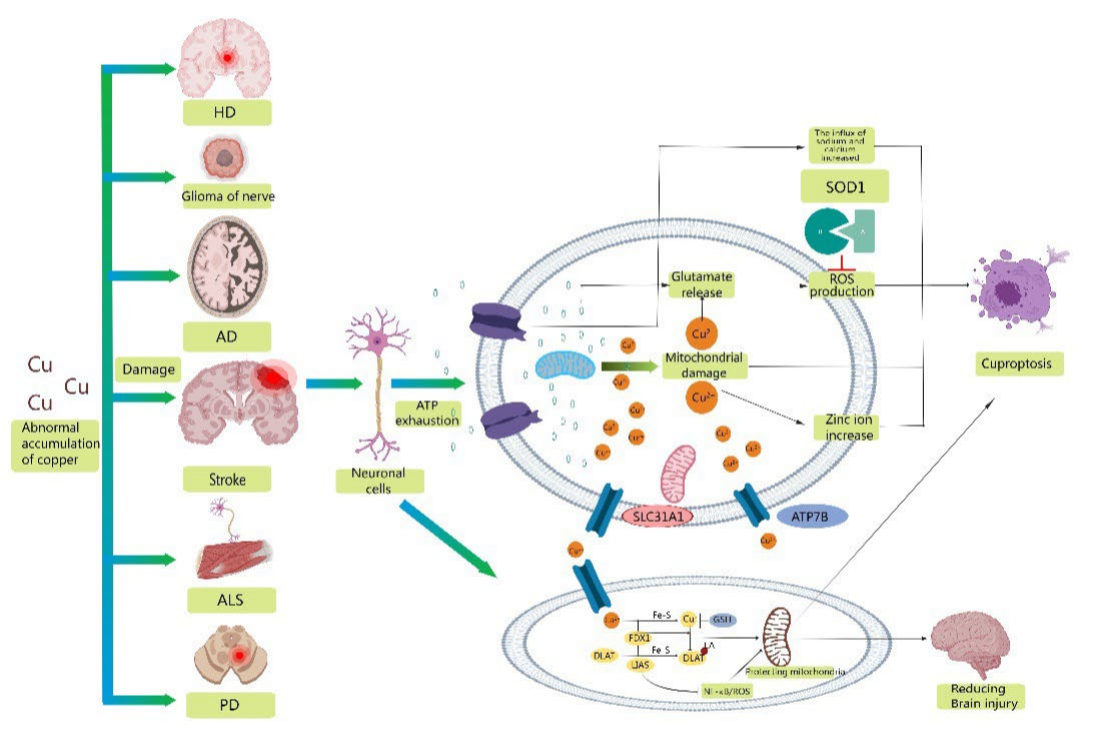
Schematic representation of possible mechanisms of cuproptosis in stroke. Abnormal accumulation of copper contributes to the development of a variety of neurodegenerative diseases, including stroke, Alzheimer's disease, amyotrophic lateral sclerosis, Parkinson's disease, glioma, and Huntington's disease. Mitochondrial oxidative stress injury is involved in the progression of stroke, providing conditions for the occurrence of cuproptosis. Therefore, reducing mitochondrial ROS production, excluding damaged mitochondria and refusing damaged mitochondria to reenter neuronal cells may be important measures to reduce neuronal oxidative stress injury and cuproptosis. Secondly, overexpression of LIAS reduced proinflammatory cytokines/chemokines and inhibited NF-κB activity. In addition, overexpression of LIAS can reduce oxidative stress and enhance antioxidant defense mechanisms, which may have some protective effect on mitochondria and reduce damage after stroke.

The importance of copper homeostasis in immune system infiltration has been shown in recent studies. TAN found that copper chelation in macrophages leads to the removal of lysyl oxidase-like 4-mediated programmed death ligand I presentation. This prevents cellular immune evasion. CHOI’s research indicated that clodoxoline, a common copper chelator, significantly reduces immune cell infiltration, including CD4 and CD8 cells, which are linked to encephalitis development (Huiqin et al., 2023). Xue suggested that the transcription factor Nrf2 and the NLRP3 inflammasome might contribute to cerebral ischemia–reperfusion injury (Fan et al., 2023). Li′s proposal was that LIAS, a protein containing Fe-S clusters, is associated with oxidative stress and inflammation (Rui et al., 2023). Overexpression of LIAS can reduce pro-inflammatory cytokines/chemokines and suppress NF-κB activity. Additionally, LIAS overexpression reduces oxidative stress and enhances antioxidant defense mechanisms, leading to an increase in the synthesis of NRF2 and LIAS. Therefore, this has the potential to safeguard mitochondrial function in diabetic nephropathy. LIAS could improve the health of stroke survivors by protecting brain mitochondria and reducing the immune response (Figure 2).

## 4 Predictions of treatment on stroke by cuproptosis

Currently, no specific drug therapy has been identified for stroke treatment through cuproptosis. However, based on the pathology of cuproptosis in stroke, certain treatment predictions have been made, summarized in Table 1.

**Table 1:** Mechanistic studies of cuproptosis and randomized controlled trials on predictions of treatment on stroke by cuproptosis.

Serial number	Predicting the direction of treatme	Methods of intervention	Groups (number of subjects)	Results	Reference
1	Mechanistic studies of cuproptosis	This paper also contains a data table for the fold changes and *p*-values of all genes analyzed in this study via a custom RT-qPCR array.	/	All compounds induced DNA damage (based on 8-oxo-guanidine, ɣH2AX staining in cells) and apoptosis (based on elevated DNA condensation/fragmentation, Annexin V staining, caspase 3/7 activity and mitochondrial membrane depolarization) in HCT-116 colon cancer cells. The increase in oxidative stress was also further confirmed in these cells.	Acilan et al. (2016)
2	Mechanistic studies of cuproptosis	In this study, we investigate the association of brain copper (assessed using ICP-MS in four regions -inferior temporal, mid-frontal, anterior cingulate, and cerebellum) and dietary copper with cognitive decline and AD pathology burden (a quantitative summary of neurofibrillary tangles, diffuse and neuritic plaques in multiple brain regions) at autopsy examination among deceased participants [*N* = 657; age of death: 90.2(±6.2) years, 70% women, 25% APOE-ɛ4 carriers] in the Rush Memory and Aging Project.	From the beginning of the MAP study in 1997 until 2017, 884 MAP participants died and had an autopsy, and 680 were consecutively analyzed for brain metal levels. Out of 680 participants, 664 had available neuropathological data, cognitive data, and brain copper data in all four regions. We excluded 7 participants with outlier values of brain copper in exactly one brain region, leaving 657 participants for analysis.	Dietary copper intake was not associated with brain copper levels or AD pathology. Associations of higher brain copper levels with slower cognitive decline and with less AD pathology support a role for copper dyshomeostasis in AD pathogenesis and suggest that lower brain copper may exacerbate or indicate disease severity. Dietary and brain copper are unrelated but dietary copper is associated with slower cognitive decline via an unknown mechanism.	Agarwal et al. (2022)
3	Mechanistic studies of cuproptosis	The differences between the IS group and the normal group as well as the correlation between the infiltrating immune cells and their functions were analyzed. The cuproptosis-related DEGs most related to immunity were screened out, and the risk model was constructed. Finally, Gene Ontology (GO) function, Kyoto Encyclopedia of Genes and Genomes (KEGG) pathway enrichment analyses and drug target were performed using the Enrichr website database. miRNAs were predicted using FunRich software. Finally, cuproptosis-related differentially expressed genes (DEGs) in IS samples were typed, and Gene Set Variation Analysis (GSVA) was used to analyze the differences in biological functions among the different types.	/	NLRP3, NFE2L2, ATP7A, LIPT1, GLS, and MTF1 may serve as predictors of cuproptosis and play an important role in the pathogenesis of immune infiltration in IS.	Fan et al. (2023)
4	Maintaining the balance of copper	This meta-analysis searched electronic databases for relevant studies on serum copper and ischemic stroke from inception through February 28, 2019, to assess the association between serum copper changes and ischemic stroke.	Eight studies with a total of 777 participants were included into this meta-analysis.	The results showed the serum copper levels were significantly higher in ischemic stroke group compared with controls group [pooled mean difference, 1.25; 95% confidence intervals (CIs), 0.07–2.43; *p* = 0.04], in particular studies after the year of 2009 (*I*^2^ = 0%; pooled mean difference, 2.16; 95% CI, 1.37–2.95; *p* < 0.00001).	Zhang et al. (2020)
5	Maintaining the balance of copper	The nearest-neighbor propensity score matching (PSM) with a ratio of 1:2 was used to reduce selection bias. The non-linear relationship was explored with restricted cubic splines (RCS). The correlation between copper intake and baseline characteristics was detected by the Pearson correlation coefficient.	A total of 10,550 participants from the 2013–2018 National Health and Nutrition Examination Survey (NHANES) were included in this study. Two 24-h dietary reviews and a standardized questionnaire were used to determine copper intake and stroke, respectively.	Subjects in the higher quartile of copper intake were significantly less likely to have a stroke compared to subjects in the first quartile of copper intake. Copper intake had a significant protective effect on women, people younger than 65 years, hypertensive patients, smokers, and diabetic stroke patients.	Yang et al. (2022)
6	Maintaining the balance of copper	In this study, a nested case–control study was conducted from the H Hypertension and Stroke Prevention and Control Program to assess the relationship between plasma copper and first stroke by conditional logistic regression analysis.	A total of 1,255 first stroke cases and 1,255 controls matched for age, sex and study site were included in the analysis	Each standard deviation (SD) increment of plasma copper was independently and positively associated with risk of first stroke [odds ratio (OR): 1.17, 95% confidence interval (CI):1.07–1.28]. The multivariable ORs with 95% CIs for total stroke, ischemic stroke and hemorrhagic stroke in the highest versus the lowest quartile of plasma copper were 1.49 (1.16–1.90; P-trend = 0.001), 1.46 (1.12–1.92; P-trend = 0.004), and 2.05 (0.95–4.38; P-trend = 0.050),respectively.	Hu et al. (2022)
7	Inhibiting cuproptosis enzymes	The main objective of this work is to determine the mechanism for misfolding and aggregation as a result of mutations in Cu-Zn superoxide dismutase1. All the structures were optimized using density functional theory (B3LYP) with 6–31G* and LANL2DZ basis sets.	/	In this study, the active site structure of SOD1 and the mutant structures of H46R, H48Q, H80R and H80G were investigated using density functional theory. The binding affinities of each residue to the Cu2+ and Zn2+ binding sites were calculated, and it was concluded that, except for H80G, the binding affinities of the other mutant structures to metal ions were low.	Keerthana and Kolandaivel (2015)
8	Inhibiting cuproptosis enzymes	The aim of this study was to detect misfolded Cu/Zn SOD1 as a potential biomarker for amyotrophic lateral sclerosis (ALS). Two ultrasensitive immunodetection assays were developed for the quantification of total and misfolded SOD1.	/	The detection of total and misfolded SOD1 was possible in human serum and cerebrospinal fluid. Total SOD1 was increased in cerebrospinal fluid from ALS patients. Misfolded SOD1 had low and variable expression in both control and ALS patient samples.	Morichon et al. (2023)
9	Inhibiting cuproptosis enzymes	This study examined whether genetically manipulated overexpression of SOD1 improves the survival of transplanted stem cells and accelerates the improvement of ischemic stroke. NSCs genetically modified to overexpress or underexpress SOD1 were placed in the brain 2 days after transient middle cerebral artery occlusion. Histological and behavioral tests were examined from day 0 to day 28 after stroke.	The purpose of this study was to determine the relationship between SOD1 expression and survival of NSCs after ischemic reperfusion injury using NSCs isolated from wild-type (WT), SOD1 transgenic (Tg), and SOD1 knockout (KO) mice.	Overexpression of SOD1 suppressed production of superoxide anions after ischemic reperfusion injury and reduced NSC death after transplantation. In contrast, downexpression of SOD1 promoted superoxide generation and increased oxidative stress-mediated NSC death. Transplantation of SOD1-overexpressing NSCs enhanced angiogenesis in the ischemic border zone through upregulation of vascular endothelial growth factor. Moreover, grafted SOD1-overexpressing NSCs reduced infarct size and improved behavioral performance compared with NSCs that were not genetically modified.	Sakata et al. (2012)
10	Inhibiting cuproptosis enzymes	In this study, we investigated whether SOD1 overexpression using gene therapy techniques in non-transgenic animals would increase neuronal survival. A neurotropic, herpes simplex virus-1 (HSV-1) vector containing the SOD1 gene was injected into the striatum either before or after transient focal cerebral ischemia.	α4s (nos-ischemic and ischemic),SOD1 (nos-ischemic and ischemic)	Striatal neuron survival at 2 days was improved by 52% when vector was delivered 12–15 h prior to ischemia and by 53% when vector delivery was delayed 2 h following ischemia.	Davis et al. (2007)
11	Cuproptosis and neural regenerati	The aim of this study was to examine the role of SOD1 overexpression in peripheral nerve regeneration and neuropathic pain-related behavior in mice. Sciatic nerves of SOD1-overexpressing and FVB/N wild type-mice were transected and immediately resutured. Evaluation of motor and sensory function and autotomy was carried out during 4 weeks of followup.	Ten young adult homozygous (Tg group) Tg mice were used for this study. Control mice (C group; n ¼ 10) were the wild-type littermates.	The findings suggest that overexpression of SOD1 is detrimental to the nerve regeneration process and exacerbates the neuropathic pain state in mice. This can be attributed, at least in part, to a disturbed inflammatory response at the site of injury.	Kotulska et al. (2006)
12	Cuproptosis and neural regenerati	Examined the effect of the bis(thiosemicarbazonato)-copper complex, CuII(gtsm) on neuritogenesis and neurite elongation (neurogenerative outcomes) in PC12 neuronal-related cultures.	5 nM FK506 (calcineurin phosphatase inhibitor),50 nM Cu^II^(gtsm)	The mechanism of neurogenerative action was investigated and revealed that CuII(gtsm) inhibited cellular phosphatase activity.Treatment of cultures with 5 nM FK506 (calcineurin phosphatase inhibitor) resulted in analogous elongation of neurites compared to 50 nM CuII(gtsm), suggesting a potential link between CuII(gtsm)-mediated phosphatase inhibition and neurogenerative outcomes.	Bica et al. (2014)
13	Cuproptosis and neural regenerati	In this study, 4-week-old male mice were exposed to Cu by free-drinking water for three months. Then, the effects of Cu on cognitive functions in mice were tested by Morris water maze tests, and the potential mechanisms were investigated by the ELISA, immunochemistry, TUNEL, and Western blot tests.	After a week of acclimatization, the mice were randomly divided into three different groups (N = 10 × 3 = 30): control group (distilled water), low-dose group (100 mg/L Cu), and high-dose group (500 mg/L Cu).	It was found that Cu exacerbates learning and memory impairment, and leads to Cu-overload in the brain and urine of mice. The results showed that Cu induces neuronal degeneration and oxidative damage, promotes the expression of apoptosis-related protein Bax, cuproptosis-related proteins FDX1 and DLAT and the proteotoxic stress marker HSP70, and decreases Fe-S cluster proteins. In addition, Cu affects the pre-synaptic and post-synaptic regulatory mechanisms through inhibiting the expression of PSD-95 and SYP. Cu also suppresses phosphorylation levels in CREB and decreases the expression of BDNF and TrkB in the mouse hippocampus.	Zhang et al. (2023)

### 4.1 Maintenance of the balance of copper ions in the brain

Copper ions play a crucial role in the process of cuproptosis, and impeding their cellular uptake could serve as a potential preventive measure against cuproptosis. Moreover, a previous study has demonstrated that the entry of copper (Cu) ions into cells can lead to their accumulation and subsequent induction of cell death (Xiong et al., 2023). Another study indicated that serum copper levels were significantly elevated in individuals with ischemic stroke compared with the control group (Zhang et al., 2020). However, a separate case–control study showed an inverse relationship between daily dietary copper intake and stroke risk (Yang et al., 2022). Hu’s research on the plasma copper levels of 1,255 individuals who experienced their first stroke found a positive association that followed a linear trend between plasma copper levels and the risk of first ischemic stroke (Hu et al., 2022). While no definitive study has yet established the efficacy of maintaining copper ion balance in the brain as a treatment for stroke, multiple sources suggest that copper ions play a significant part in the stroke process.

### 4.2 Enzymes that inhibit cuproptosis

Cuproptosis involves several distinct enzymes that are promising therapeutic targets for stroke management (Robinson and Winge, 2010). The enzyme Cu/Zn-SOD1 is a dimeric cytosolic enzyme (Keerthana and Kolandaivel, 2015). A study investigated the potential of misfolded Cu/Zn-SOD1 as a biomarker for ALS by detecting total SOD1 and misfolded SOD1 in human serum and cerebrospinal fluid (Morichon et al., 2023). ALS, a neurodegenerative disorder, shares some pathophysiological similarities with those of stroke (Mohr, 2004). Sakata’s study concluded that the overexpression of SOD1 could decrease ROS levels, enhance neural stem cell survival, and protect the mouse brain during temporary focal cerebral ischemia episodes (Sakata et al., 2012). Alexis’s research showed that upregulating SOD1 using a herpes simplex virus vector could provide neuroprotection to striatal neurons during temporary focal cerebral ischemia (Davis et al., 2007) episodes. Thus, it is plausible to hypothesize that inhibiting these enzymes could help prevent or mitigate cuproptosis, potentially improving the prognosis for individuals affected by stroke.

### 4.3 Cuproptosis and nerve regeneration

A study suggests that copper ions may inhibit neuron growth and repair, adversely impacting nerve regeneration (Kotulska et al., 2006). Conversely, copper ions could have a beneficial effect on this process. Various studies indicate that copper ions aid axon growth and synapse formation, contributing to brain function restoration (Bica et al., 2014). The CREB/BDNF pathway plays a crucial role in nerve regeneration. Research indicates that (Zhang et al., 2023) copper can disrupt synaptic plasticity through the CREB/BDNF pathway, trigger cuproptosis, and promote cell death, suggesting a strong link between copper-induced cell death and nerve regeneration. Additionally, cuproptosis’s influence on nerve regeneration is complex, as it can either support or hinder repair. This effect is similar to neuroinflammation post-stroke, where neuroinflammation can either aid recovery or intensify brain damage (Stuckey et al., 2021). Thus, determining the optimal timing for intervening in neuroinflammation is vital. This insight leads to the hypothesis that modulating cuproptosis timing in nerve regeneration could allow control over the regenerative process.

## 5 Summary and outlook

Given the accumulation of copper in various neurodegenerative diseases, it’s plausible that cuproptosis plays a pathogenic role, potentially offering a new therapeutic target. Strategies such as using copper chelators, reducing dietary copper intake, and genetically modifying copper transporters can mitigate copper-induced cell death by lowering intracellular copper levels. However, further research is needed to fully understand how cuproptosis contributes to disease progression.

The lipoic acid pathway is key in mediating cuproptosis, but the exact mechanisms of this cell death form remain unclear, including the role of other metabolic pathways and how lipoylated mitochondrial enzyme aggregation triggers copper-dependent cell death. Further investigation is necessary to uncover these mechanisms and explore clinical applications. Recent studies highlight the significant role of cuproptosis in stroke progression and provide new opportunities for clinical applications.

Despite the ongoing uncertainty surrounding the exact mechanism of cuproptosis in relation to stroke, a growing body of experimental research has highlighted the significant role of cuproptosis in stroke progression (Fan et al., 2023). At present, the main approaches to stroke management include pharmacotherapy, interventional procedures, surgical interventions, and rehabilitation. However, these treatments have inherent limitations. The link between cuproptosis and stroke offers promising opportunities for improving stroke treatments, emphasizing the need for targeted research into the effects of cuproptosis on stroke.

## Author contributions

LX: Writing – original draft, Writing – review & editing. ZW: Data curation, Methodology, Writing – original draft. ZH: Data curation, Writing – original draft. PP: Conceptualization, Investigation, Writing – review & editing. AY: Methodology, Writing – review & editing. JW: Conceptualization, Writing – review & editing.
